# The role of sleep state and time of day in modulating breathing in epilepsy: implications for sudden unexpected death in epilepsy

**DOI:** 10.3389/fncir.2022.983211

**Published:** 2022-08-23

**Authors:** Katelyn G. Joyal, Benjamin L. Kreitlow, Gordon F. Buchanan

**Affiliations:** ^1^Interdisciplinary Graduate Program in Neuroscience, Carver College of Medicine, University of Iowa, Iowa City, IA, United States; ^2^Department of Neurology, Carver College of Medicine, University of Iowa, Iowa City, IA, United States; ^3^Iowa Neuroscience Institute, Carver College of Medicine, University of Iowa, Iowa City, IA, United States; ^4^Medical Scientist Training Program, Carver College of Medicine, University of Iowa, Iowa City, IA, United States

**Keywords:** epilepsy, SUDEP, sleep, circadian, breathing

## Abstract

Sudden unexpected death in epilepsy (SUDEP) is the leading cause of death among patients with refractory epilepsy. While the exact etiology of SUDEP is unknown, mounting evidence implicates respiratory dysfunction as a precipitating factor in cases of seizure-induced death. Dysregulation of breathing can occur in epilepsy patients during and after seizures as well as interictally, with many epilepsy patients exhibiting sleep-disordered breathing (SDB), such as obstructive sleep apnea (OSA). The majority of SUDEP cases occur during the night, with the victim found prone in or near a bed. As breathing is modulated in both a time-of-day and sleep state-dependent manner, it is relevant to examine the added burden of nocturnal seizures on respiratory function. This review explores the current state of understanding of the relationship between respiratory function, sleep state and time of day, and epilepsy. We highlight sleep as a particularly vulnerable period for individuals with epilepsy and press that this topic warrants further investigation in order to develop therapeutic interventions to mitigate the risk of SUDEP.

## Introduction

Epilepsy is one of the most common neurological disorders. One in 26 Americans will develop epilepsy during their lifetime (Kotsopoulos et al., 2002 ; Hesdorffer et al., 2011). Despite its prevalence, approximately 35% of patients will not achieve seizure freedom with medical treatment (Kwan and Brodie, 2000; Chen et al., 2018). Though there has been continued expansion in the availability of anti-seizure medications (ASM), patients who exhibit an inadequate response to initial ASM treatment are likely to have medically refractory epilepsy (Kwan and Brodie, 2000). The leading cause of death among these individuals with poor seizure control is sudden unexpected death in epilepsy or SUDEP (Devinsky et al., 2016). SUDEP is defined as the “sudden, unexpected, witnessed or unwitnessed, nontraumatic and nondrowning death in patients with epilepsy, with or without evidence for a seizure and excluding documented status epilepticus, in which postmortem examination does not reveal a toxicologic or anatomic cause of death” (Nashef et al., 1998). While by definition SUDEP does not have to follow a seizure, there is strong evidence to suggest it is a seizure-related phenomenon, with its agonal mechanisms beginning during or in the immediate aftermath of a seizure (Nashef et al., 1998; Nilsson et al., 1999; Surges et al., 2009; Surges and Sander, 2012; Bozorgi and Lhatoo, 2013). There is a slight predominance of SUDEP cases in males compared to females (Tennis et al., 1995; Nilsson et al., 1999; Shankar et al., 2013).

Despite the tremendous burden of SUDEP, its underlying pathological mechanisms are poorly understood. However, evidence is accumulating that implicates seizure-related respiratory failure as a major factor in this deadly phenomenon (Ryvlin et al., 2013; Buchanan et al., 2014; Kim et al., 2018; Dhaibar et al., 2019). In SUDEP cases that were captured in epilepsy monitoring units (EMU), terminal apnea preceded terminal asystole in every case (Ryvlin et al., 2013). Further, mechanical ventilation has been found to greatly reduce seizure-induced mortality, both in human patients and animal models (Tupal and Faingold, 2006; Ryvlin et al., 2013; Buchanan et al., 2014). Thus, further investigation into respiratory dysfunction in epilepsy is critical to untangle the underlying mechanisms of SUDEP, as well as to assist clinicians in developing respiratory-focused interventions.

Another consistent observation is that SUDEP cases predominantly occur during the night (Nobili et al., 2011; Lamberts et al., 2012; Sveinsson et al., 2018). Around 95% of SUDEP cases occur inside the victim’s residence, with the majority of victims found in or near a bed in a prone position (Opeskin and Berkovic, 2003; Zhuo et al., 2012; Ali et al., 2017; Sveinsson et al., 2018). Despite occurring so close to home, the vast majority of these cases are unwitnessed (Lamberts et al., 2012; Zhuo et al., 2012; Rugg-Gunn et al., 2016; Purnell et al., 2018). Patients who die of SUDEP are twice as likely to have a history of nocturnal seizures, and thus the presence of nocturnal seizures are considered a risk factor for SUDEP (Lamberts et al., 2012; Shankar et al., 2013; Sveinsson et al., 2018; Van Der Lende et al., 2018). Seizures and epileptiform discharges occur more frequently during non-rapid eye movement (NREM) sleep in both human patients and animal models (Bazil and Walczak, 1997; Malow et al., 1998). Sleep state can influence the frequency, severity, and duration of seizures (Bazil and Walczak, 1997; Ng and Pavlova, 2013). Seizures occurring during sleep tend to be longer and are more likely to evolve into focal and bilateral tonic-clonic seizures (Bazil and Walczak, 1997).

**Figure 1:**
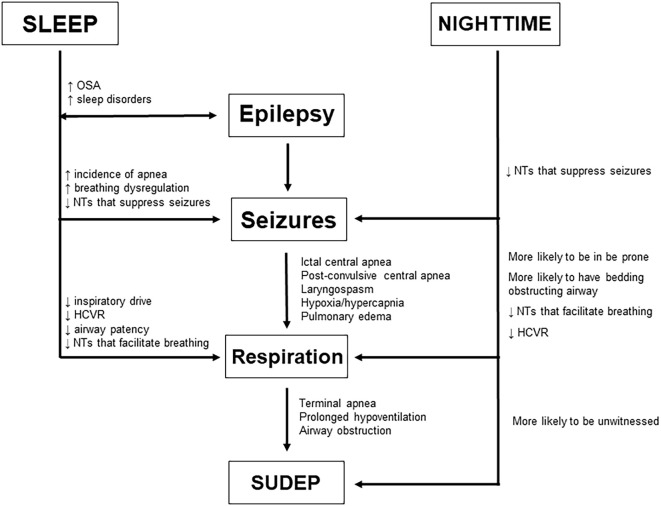
Potential risk factors associated with seizures emerging from sleep vs. nocturnal seizures and how they may facilitate SUDEP by modulating epilepsy, seizures, and respiration as well as seizure-induced death itself.

As humans tend to consolidate their sleep during the night, many investigations of and conclusions about SUDEP risk factors conflate sleep-state and nighttime as one in the same. In reality, sleep and circadian rhythmicity can independently alter physiological processes, including respiratory and cardiac function (Snyder et al., 1964; Browne et al., 1983; Spengler et al., 2000; Mortola, 2004; Buchanan, 2013). The major influence of sleep and circadian timing on respiration makes this a salient point of examination when considering SUDEP pathophysiology. The aim of this review is to examine the distinct influences of sleep and circadian rhythms on respiration both in a healthy brain and in patients with epilepsy (Figure 1). We hope to not only highlight the factors that make nocturnal seizures more deadly, but to better differentiate between sleep-state and time-of-day influences on breathing, so that clinicians can develop specific preventative strategies for fatal seizure-induced respiratory dysfunction.

## Influence of Sleep on Breathing

**Figure 2:**
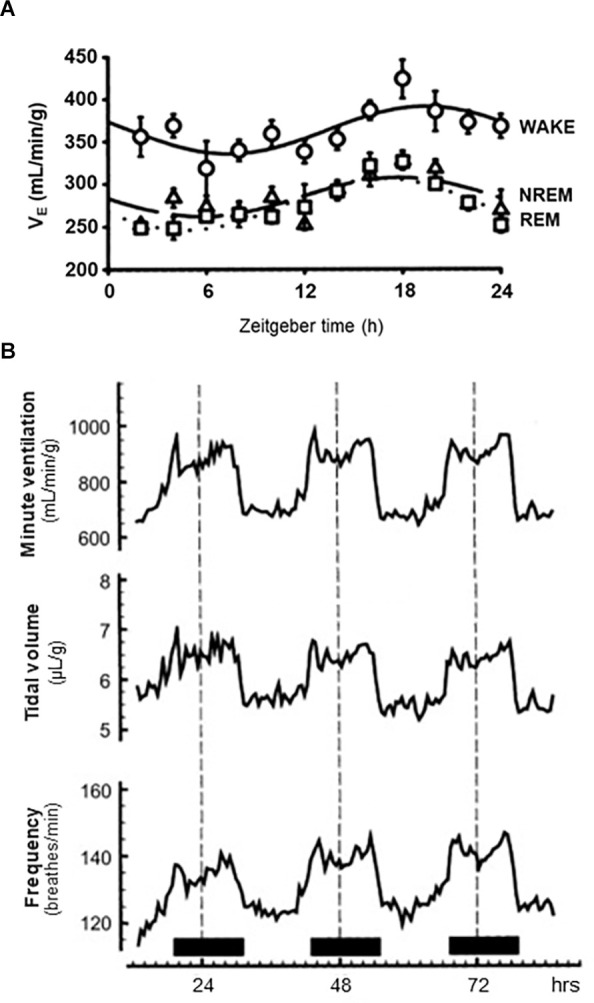
Circadian and sleep state-dependent effects on ventilation. **(A)** 72-h traces of average minute ventilation (top), tidal volume (middle) and breathing frequency (bottom) in adult male rats housed under a 12:12 h light:dark cycle and receiving room air (21% O_2_, balance N_2_). Solid horizontal bars at the bottom indicate periods where lights were off. **(B)** 24-h trace of average minute ventilation in rats during wake, non-rapid eye movement (NREM) sleep, and rapid-eye movement (REM) sleep as indicated. All animals housed in a 12:12 h light:dark cycle. **(A)** Redrawn with permission from Seifert and Mortola (2002). **(B)** Redrawn with permission from Stephenson et al. (2001).

**Figure 3:**
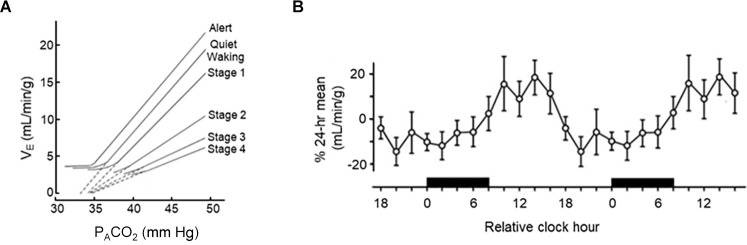
Circadian and sleep state-dependent effects on the hypercapnic ventilatory response (HCVR). **(A)** 48-h trace of circadian variations in HCVR in adult humans. **(B)** Sleep state-dependent differences in HCVR in adult males. **(A)** Redrawn with permission from Spengler et al. (2000). **(B)** Redrawn with permission from Bulow (1963).

It has long been appreciated that breathing is regulated in a sleep state-dependent manner (Snyder et al., 1964; Spengler et al., 2000; Haxhiu et al., 2003; Mortola, 2004; Malik et al., 2012; Buchanan, 2013). Inspiratory drive is lower during NREM sleep and lowest during rapid-eye movement (REM) sleep, with tidal volume (V_T_) being reduced to 73% of its level during wakefulness (Douglas et al., 1982a; Figure 2A). Within NREM sleep, the nadir of minute ventilation (V_E_) occurs during NREM stage 3 (N3) sleep—although this is likely driven by the reduction in V_T_. This results in an end-tidal carbon dioxide (ETCO_2_) concentration that is 1–2 torr higher than waking levels (Krieger, 2005). This drop in V_T_ and V_E_ is likely due to decreased chemosensitivity during the onset of sleep (Bulow, 1963; Douglas et al., 1982b, c). During sleep there is a decrease in the respiratory response to hypercapnia (Reed and Kellogg, 1958; Birchfield et al., 1959; Cherniack, 1981; Douglas et al., 1982c; Berthon-Jones and Sullivan, 1984; Figure 3A) as well as hypoxia (Berthon-Jones and Sullivan, 1982; Douglas et al., 1982b; Malik et al., 2012). Like inspiratory drive, there is an even larger decrease in the hypoxia-induced respiratory drive during REM compared to NREM sleep (Berthon-Jones and Sullivan, 1984; Malik et al., 2012). There are sex-specific differences in the response to hypercapnia, with males exhibiting a 50% decrease in the hypercapnic ventilatory response (HCVR) compared to wakefulness, while females exhibit a reduced HCVR during wakefulness compared to males but have less apparent reductions in the response during sleep (Berthon-Jones and Sullivan, 1984). Progesterone has been found to stimulate breathing during sleep, including increasing hypoxic and hypercapnic respiratory responses (Javaheri and Guerra, 1990; Saaresranta et al., 1999). Progesterone oscillates in a circadian fashion, with its zenith at around midnight (Junkermann et al., 1982; Gharib et al., 2018). No sex-specific differences in respiratory responses to hypoxia have been identified (Malik et al., 2012).

Breathing during NREM sleep has a more regular pattern compared to breathing during wakefulness, without altering mean breathing frequency (Malik et al., 2012). Conversely, during REM sleep there is more variability in respiratory patterns, including increased frequency, decreased regularity, and brief periods of central apnea (Aserinsky and Kleitman, 1953; Cherniack, 1981; Malik et al., 2012). There is some evidence that indicates this irregular breathing is a response to cortical inputs that reflect the content of the individual’s dream (Oudiette et al., 2018). Periodic breathing, which is characterized as clusters of breaths separated by intervals of central apnea or near apnea, also sometimes occurs during sleep. Although previously thought to arise from a severe neurological or cardiovascular condition, it now found that periodic breathing can occur in healthy individuals, especially during hypoxia (Berssenbrugge et al., 1983; Cherniack, 1999; Ainslie et al., 2013). During intervals of periodic breathing, cyclic changes in ventilation as well as the partial pressures of carbon dioxide (CO_2_) and oxygen (O_2_) can trigger oscillations in heart rate, blood pressure, autonomic nervous system activity, and upper-airway resistance. This may create a feedback loop whereby these oscillations in turn affect ventilation and increase the length and symmetry of these periodic breathing cycles (Cherniack, 1999). Males tend to exhibit periodic breathing in response to hypoxia more frequently than females (Pramsohler et al., 2019). Breathing patterns are heavily dependent on the pre-Bötzinger complex (pre-BötC; Smith et al., 1991; Buchanan, 2013; Del Negro et al., 2018; Muñoz-Ortiz et al., 2019). When neurons expressing neurokinin-1 receptors (NK1R) in the pre-BötC complex were bilaterally ablated in adult rats there was a progressive and irreversible disruption in breathing stability, which initially occurred only during sleep, but eventually led to ataxic breathing during wakefulness as well (Mckay and Feldman, 2008). When these pre-BötC NK1R-expressing neurons were unilaterally ablated, there was a disruption in respiratory pattern and increase in the frequency of central sleep apnea and hypopneas solely during sleep, particularly during REM sleep, which never developed during wakefulness (Mckay and Feldman, 2008).

During sleep there is a reduction in upper airway patency and an increase in respiratory resistance. This is caused by a preferential reduction in tone of laryngeal and pharyngeal muscles that help to maintain the structure of the upper airway (Cherniack, 1981; Haxhiu et al., 1987; Buchanan, 2013; Kubin, 2016). This reduced patency and can be especially problematic during REM sleep, when breathing is particularly unstable (Cherniack, 1981). Upper airway tone is controlled by inputs from trigeminal (CN V), facial (CN VII), and hypoglossal (CN XII) motor neurons (Buchanan, 2013). The genioglossus muscle, which is innervated by hypoglossal motor neurons, is the largest and most extensively studied of the airway dilator muscles. It has been suggested that decreased serotonergic and noradrenergic inputs to hypoglossal motor neurons during REM sleep causes atonia of the genioglossus (Fenik et al., 2005). The genioglossus and other muscles of the upper airway require both tonic and phasic inspiratory activation in order to protect against collapse (Kubin, 2016). When the tone of these airway-dilating muscles can no longer oppose the negative inspiratory pressure, the result is obstructive sleep apnea (OSA), which features recurrent episodes of hypopneas and apneas (Remmers et al., 1978; Kubin, 2016). While these obstructive apneas only occur during sleep, frequent sleep apnea and hypoventilation can result in breathing abnormalities during wakefulness (Simonds, 1994).

Changes in several non-centrally mediated respiratory mechanisms are also associated with the onset of sleep. During NREM sleep, the activity of the intercostal muscles is increased compared to wakefulness (Malik et al., 2012). This may be indicative of increased contribution of the chest wall to respiration in order to compensate for decreased central inspiratory drive. During REM sleep, there is a loss of tonic activity in the intercostals and diaphragm (Tusiewicz et al., 1977; Bryan and Muller, 1980; Malik et al., 2012). Chest wall compliance is also increased during this time, and, in conjunction with decreased intercostal tone, can cause paradoxical collapse of the chest during inspiration (Malik et al., 2012). Lastly, the pulmonary stretch receptor reflex and irritant receptor reflex are suppressed during sleep—thus, coughing in response to apnea only occurs after arousal (Douglas, 2000). In summary, sleep is a period where many facets of breathing are suppressed—thus rendering it a particularly vulnerable period for further insults to the respiratory system.

## Circadian Influences on Breathing

Early studies of time-of-day variability in mammalian (adult rat) breathing physiology revealed time-of-day differences in breathing; however, the effect was limited to CO_2_ production and the mean inspiratory air flow (Peever and Stephenson, 1997). Under hypercapnic conditions, breathing frequency and V_E_ also appeared to be time-of-day dependent. Unfortunately, these studies only involved two time-points, limiting the resolution of a daily rhythm, which may have been masked by higher frequency ultradian variation in breathing (Stupfel and Pletan, 1983; Stupfel et al., 1985).

The first clear evidence that respiratory function demonstrated daily oscillations came from Seifert et al. (2000). Adult rats were housed in 10 L barometric chambers with carefully controlled in-flow and out-flow of gas, allowing for measurement of breathing physiology over the course of several days. Clear time-of-day differences in frequency, V_T_ and V_E_ were observed. The highest levels were observed during the dark phase, coinciding with elevated temperature and activity. These findings were further expanded in a later study, demonstrating that O_2_ consumption (a measure of metabolic activity), inspiratory time, and expiratory time also varied across the day (Seifert and Mortola, 2002; Figure 2B). Interestingly, controlling for level of activity did not eliminate the effect of time of day on V_E_, V_T_, frequency of breathing, or O_2_ consumption. The authors conclude that the daily variability observed in breathing (specifically ventilation) is likely driven by other physiological variables oscillating throughout the day, such as temperature and oxygen consumption.

Using similar methods to Seifert et al., 2000, long-term respiratory monitoring in non-human primates has also been performed (Iizuka et al., 2010). Whole body plethysmography was performed in 11 unrestrained, unanesthetized male cynomolgus monkeys. Like findings from adult rats, multiple respiratory parameters, including respiratory rate, V_T_, and V_E_ were shown to vary depending on the time of day. However, recordings were only obtained hourly, and it is unclear if sleep-state was controlled for.

Time-of-day variability in a number of respiratory parameters has also been demonstrated in humans (Spengler and Shea, 2000; Spengler et al., 2000). In a carefully controlled laboratory setting, which included the removal of external time cues (except for lighting), a constant environmental temperature, controlled dietary intake, and carefully controlled sleep schedules. Temporal variation in rectal temperature and plasma cortisol were used as endogenous circadian markers. ETCO_2_, O_2_ consumption, and CO_2_ production were all shown to oscillate throughout the 24 h day, with highest levels in the morning. Interestingly, there was also time-of-day variability in HCVR magnitude, a finding previously demonstrated in awake, adult rats (Peever and Stephenson, 1997; Figure 3B), suggesting respiration-influencing chemosensitivity may also be under circadian regulation. Sensitivity to isocapnic hypoxic challenge has some evidence of time-of-day dependence; however, the effect is far less pronounced (Siekierka et al., 2007).

While the studies described above in rats, monkeys, and humans demonstrate temporal variation in breathing physiology, they did not control for the rhythmic effect of light. Therefore, whether this variability is due to the effect of an external time cue or that of an endogenous circadian rhythm cannot be concluded. The first study of time-of-day dependent on breathing physiology that accounted for the influence of light was performed in garter snakes (Hicks and Riedesel, 1983). Animals were housed in either a 14:10 light-dark cycle or constant darkness environment. Under these conditions, it was revealed that time-of-day variability in oxygen consumption, breathing frequency, V_T_, and V_E_ persisted in constant darkness, suggesting endogenous regulation of these breathing parameters.

This time-of-day dependent variation in breathing has been shown to be endogenously circadian in mice and mediated by the body’s central circadian pacemaker, the suprachiasmatic nucleus (Purnell and Buchanan, 2020). C57BL/6J mice were housed in either 12:12 light-dark or constant darkness environments, and running wheels were used to assess active phase onset for the determination of individual free-running locomotive rhythms. As sleep-state has been shown to influence breathing (as described in detail earlier above), measurements of breathing were only performed while the animals were awake. Time-of-day variability in the frequency of breathing and V_E_, but not V_T_, was observed. Both frequency and V_E_ were highest during the dark phase of the day. This time-of-day rhythm was shown to be circadian, as these two rhythms persisted when animals were housed in constant darkness. Electrolytic lesioning of the suprachiasmatic nucleus eliminated these breathing rhythms, suggesting that the circadian variation in breathing was controlled by the suprachiasmatic nucleus.

Although the suprachiasmatic nucleus is frequently referred to as the master circadian oscillator, nuclei outside of the suprachiasmatic nucleus and peripheral tissues may contain autonomous circadian clocks (Mohawk et al., 2012). Such peripheral clocks have been described in brainstem and spinal cord neurons involved in the coordination and output necessary to maintain normal respiration. Through the measurement of molecular clock gene transcripts, such as *Clock*, *Bmal1*, and *Per1/2*, researchers have identified robust molecular clock gene rhythms in the nucleus tractus solitarius (Kaneko et al., 2009; Chrobok et al., 2020), phrenic motor nucleus (Kelly et al., 2020), and laryngeal, tracheal, bronchial, and lung tissues within the airway (Bando et al., 2007). Bando et al. (2007) demonstrated that the peripheral clock of the airway tissues could be rendered arrhythmic following electrolytic lesioning of the suprachiasmatic nucleus. Similarly, genetically arrhythmic *Clock 1/Clock2* knock-out mice did not demonstrate peripheral rhythmicity of clock gene expression in airway tissues. In conclusion, circadian phase exerts its own powerful influences on breathing, irrespective of vigilance state.

## Effect of Seizures/Epilepsy on Breathing

Some patients with epilepsy experience breathing abnormalities at baseline which may be further compromised during a seizure. Sainju et al. found a blunted hypercapnic ventilatory response in a subset of patients with epilepsy, placing them at greater risk for peri-ictal hypoventilation (Sainju et al., 2019). Patients with Dravet syndrome (DS) similarly exhibit a decreased ventilatory response to CO_2_ (Kim et al., 2018). Several animal models of epilepsy present with respiratory dysregulation, even in the absence of a seizure. The *Kcna1*-null mutant model exhibits progressive respiratory dysfunction with age (Simeone et al., 2018). Like their human counterparts, the *Scn1a^R1407X/+^* human knock-in mouse model of DS has a diminished ventilatory response to CO_2_, as well as baseline hypoventilation and apnea (Kuo et al., 2019). A similar loss of the hypercapnic ventilatory response has been found in animals that have undergone amygdala kindling (Totola et al., 2019). Hajek and Buchanan (2016) found that mice with increased respiratory rate variability at baseline are more likely to die following a maximal electroshock (MES) seizure. These findings support the idea that interictal respiratory dysfunction may serve as a biomarker for those at greater risk for SUDEP.

Seizures themselves can cause profound alterations in respiration, including coughing, apnea, hyperventilation, bronchial spasms, increased pulmonary vascular pressure, laryngospasm, and pulmonary edema (Bayne and Simon, 1981; Kennedy et al., 2015; Nakase et al., 2016; Rugg-Gunn et al., 2016). Seizures appear to cause varying degrees of respiratory dysregulation depending on seizure type and origin (Bateman et al., 2008; Blum, 2009). Longer duration of seizures is associated with a greater degree of dysfunction, particularly in regard to hypercapnia, pulmonary pressure, and pulmonary edema (Bayne and Simon, 1981; Bateman et al., 2008; Seyal et al., 2010; Kennedy et al., 2015).

Hypoventilation during a seizure may occur due to airway obstruction or dysregulation of the brain’s respiratory centers and usually results in hypercapnia and hypoxemia (Rugg-Gunn et al., 2016). Dravet syndrome patients in particular demonstrate peri-ictal hypoventilation, which precedes the onset of bradycardia (Kim et al., 2018). Hypoventilation can lead to secondary cardiac failure, especially during seizures where oxygen saturation (SaO_2_) drops below 90% (Seyal et al., 2011). A cause of some ictal hypoventilation is central apnea. Ictal central apnea (ICA) is a relatively frequent occurrence during seizures, especially ones with bihemispheric involvement (Nashef et al., 1996; Rugg-Gunn et al., 2016). ICA occurs exclusively in focal epilepsy, emerging during 33–50% of focal seizures (Lacuey et al., 2018; Vilella et al., 2019; Tio et al., 2020). ICA can precede electrographic seizure activity as well as clinical seizure onset by up to 7–10 s (Nishimura et al., 2015; Tio et al., 2020). These apneas tend to be brief and do not substantially impact O_2_ saturation (Bateman et al., 2008). A multivariate analysis indicated that contralateral seizure spread and seizure duration mutually contribute to increased ETCO_2_ that follows ICA (Seyal et al., 2010). Several animal models of epilepsy and SUDEP exhibit ICA, including *Scn1a^R1407X/+^* mice, in which mechanical ventilation can prevent fatal seizure-induced respiratory arrest (Kim et al., 2018). Additionally, a model of status epilepticus induced in sheep features ICA and hypoventilation in 100% of the animals, with some resulting in death (Johnston et al., 1997). Post-convulsive central apnea (PCCA), in contrast, occurs in both focal and generalized epilepsies, suggesting a separate pathophysiology from ICA (Vilella et al., 2019). PCCA is less common than ICA—occurring during only 18% of generalized seizures. However, PCCA may be much more dangerous than ICA. PCCA is associated with a longer recovery time from hypoxemia, and it is considered by some to be a biomarker for SUDEP (Jin et al., 2017; Vilella et al., 2019).

Seizures may impair a person’s ability to autoresuscitate after central apnea. Autoresuscitation is a spontaneous protective cardiorespiratory phenomenon which promotes the recovery of normal breathing and heart rate after primary apnea by initiating a gasping reflex (Adolph, 1969; Guntheroth and Kawabori, 1975). Failure to autoresuscitate has been documented in infant deaths that were eventually classified as sudden infant death syndrome (SIDS; Meny et al., 1994; Sridhar et al., 2003). There are numerous parallels between SIDS and SUDEP, including normal autopsy, prone position, predominance during the nighttime, predicted respiratory mechanism, and evidence of serotonergic system dysfunction (Richerson and Buchanan, 2011; Buchanan, 2019).

Obstructive apnea, or laryngospasm, is another seizure-associated phenomenon that can result in death (Stewart, 2018). DBA/2 mice, which display lethal audiogenic seizures, have a significantly reduced mortality rate following seizures after being implanted with a tracheal T-tube as a surrogate airway (Irizarry et al., 2020). Seizures induced *via* kainic acid in rats have been documented to cause partial or complete glottic closure and subsequent death (Nakase et al., 2016; Budde et al., 2018; Jefferys et al., 2019). It has been postulated that fatal obstructive apnea is a consequence of bronchial spasms or hypotonia of the muscles involved in respiration (Stöllberger and Finsterer, 2004). Nakase et al. proposed that ictal laryngospasm is caused by the spread of a seizure *via* autonomic medullary motor regions to the laryngeal branches of the vagus nerve (Nakase et al., 2016).

Spreading depolarization (SD) may be one of the underlying mechanisms behind cardiorespiratory failure in SUDEP. In *Cacna1a^S218L^* mutant mice, which carry a gain of function mutation in the Ca_v_2.1 voltage-gated calcium channel, brainstem SD occurs during all spontaneous fatal seizures, as well as a subset of nonfatal seizures (Jansen et al., 2019). Additionally, seizure-related SD in the ventrolateral medulla is correlated with the incidence of respiratory suppression (Jansen et al., 2019). Chemically induced seizures in *Kcna1* and *Scn1a* mutant mice cause a wave of SD in the dorsal medulla, which may temporarily silence the cells that would serve to reoxygenate the brain following a seizure (Aiba and Noebels, 2015). This depolarizing blockade may cause a positive feedback loop in which the brain cannot reoxygenate following a seizure during which oxygen saturation has dropped dramatically, potentially leading to complete cardiorespiratory arrest (Aiba and Noebels, 2015).

Numerous other potential mechanisms underlying ictal respiratory dysfunction and failure have been proposed. A leading hypothesis is that seizures activate inhibitory subcortical projections to brainstem respiratory centers (Dlouhy et al., 2015; Lacuey et al., 2017). It has also been found that central apnea occurs in human patients when seizures spread to the amygdala (Dlouhy et al., 2015; Rhone et al., 2020). Similarly, stimulation of the amygdala as well as the hippocampus produces central apnea that patients are not aware of (Dlouhy et al., 2015; Lacuey et al., 2017; Nobis et al., 2018), and they are able to voluntarily initiate inspiration when prompted (Dlouhy et al., 2015). Further studies revealed that stimulation of the basal amygdala in particular (including the basomedial and basolateral nuclei) was particularly likely to cause apnea, while stimulation of more lateral regions produced fewer apneas (Rhone et al., 2020). In DBA/1 mice, unilateral lesions to the amygdala was sufficient to suppress seizure-induced respiratory arrest (S-IRA) (Marincovich et al., 2021). This suggests that apnea is due to the loss of involuntary ventilatory drive rather than an issue with the respiratory motor output pathways or musculature.

Despite the implications of both respiratory and cardiac dysfunction contributing to SUDEP, recent evidence has surfaced suggesting respiratory failure precedes cardiac failure during instances of seizure-induced death. In 2013, a multi-center MORTality in EMUs Study (MORTEMUS) of SUDEP incidents in EMUs found that all recorded cases of SUDEP featured terminal respiratory arrest prior to terminal asystole (Ryvlin et al., 2013). Similar results have been found in the *Kcna1*-null mouse model (Dhaibar et al., 2019) and in an MES model (Buchanan et al., 2014). Another indicator of respiratory failure’s pivotal role in SUDEP is that mechanical ventilation, if administered immediately, can greatly reduce mortality in both human patients and animal models (Tupal and Faingold, 2006; Ryvlin et al., 2013; Buchanan et al., 2014). In a similar vein, oxygenation prior to seizure induction can prevent fatal audiogenic seizures in several strains of audiogenic mice, without impacting seizure incidence or severity (Venit et al., 2004). To summarize, seizures cause profound alterations in breathing which may directly contribute to seizure-induced death.

## Sleep and Circadian Effects of Seizures/Epilepsy on Breathing

**Figure 4:**
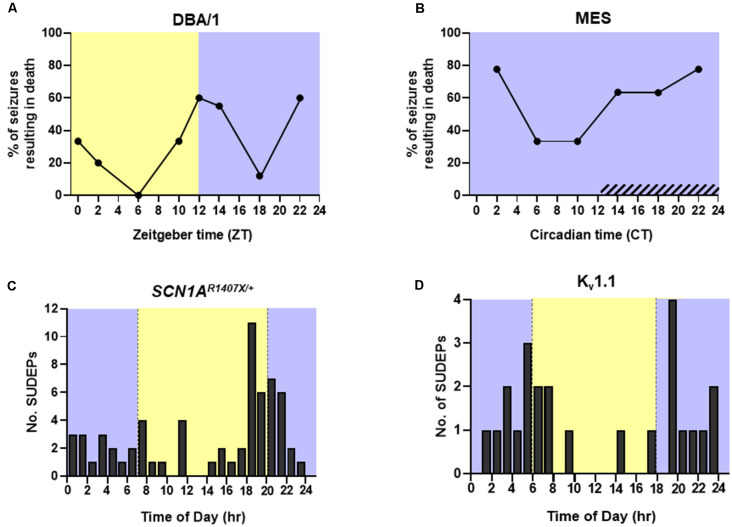
Time-of-day and circadian probability of seizure-induced death in mouse models of epilepsy. Temporal distribution of spontaneous seizure-induced death in **(C)** *SCN1A^R1407X/+^* and **(D)** Kv1.1 knockout mice housed in a 12:12 h light:dark cycle. **(A)** Percentage of audiogenic seizures resulting in death in DBA/1 mice housed in a 12:12 h light:dark cycle. **(B)** Percentage of maximal electroshock (MES) seizures resulting in death in mice housed in constant darkness. Redrawn with permission from **(C)** Teran et al. (2019), **(D)** Moore et al. (2014), **(A,B)** Purnell et al. (2021b).

Approximately 10–15% of epilepsy patients have seizures solely or primarily during sleep (Grigg-Damberger and Foldvary-Schaefer, 2021). Seizures occurring during sleep tend to be longer and are more likely to evolve into focal and bilateral tonic-clonic seizures (Bazil and Walczak, 1997). As mentioned above, longer convulsive seizures are associated with an increased degree of respiratory dysfunction (Bayne and Simon, 1981; Bateman et al., 2008; Seyal et al., 2010; Kennedy et al., 2015). Seizures emerging from sleep are also more likely to be associated with the presence of post-ictal generalized EEG suppression (PGES) and greater oxygen desaturation (Latreille et al., 2017). A clinical study in 20 patients with epilepsy found that 44% of nocturnal seizures are associated with ICA, and although the difference did not reach significance, a smaller fraction of wake-related seizures were accompanied by ICA (28%; Latreille et al., 2017). MES seizures in mice that are induced during NREM sleep are also associated with greater respiratory dysfunction than those induced during wakefulness (Hajek and Buchanan, 2016). When factoring in time of day, MES seizures that were induced during the day, the rodent inactive phase, resulted in a greater degree of postictal respiratory and EEG suppression than those induced during the nighttime. This effect was even greater when the seizures were induced during this time while the animal was in NREM sleep (Purnell et al., 2017). When DBA/1 mouse model of audiogenic seizures were exposed to an audiogenic stimulus during the day, the ensuing seizures resulted in death during 21.7% of trials. Conversely, seizures induced during the night resulted in seizure-induced death in 46.7% of trials (Purnell et al., 2021b; Figure 4A). The same study used mice living in constant darkness to access circadian influence on seizure-induced death in the MES mouse model. They found that during the subjective night there was a decrease in postictal ventilation and an increase in the probability of seizure-induced death without altering seizure severity (Purnell et al., 2021b; Figure 4B). K_v_1.1 potassium channel knockout (KO) mice and *SCN1A*^R1407X/+^ mice, which experience progressive breathing dysregulation (Kim et al., 2018; Kuo et al., 2019; Iyer et al., 2020), also experience seizure-induced death more commonly during the nighttime (Figures 4C,D).

Because humans typically sleep during the night, nighttime seizures are often unwitnessed (Lamberts et al., 2012; Zhuo et al., 2012; Rugg-Gunn et al., 2016; Purnell et al., 2018). Lamberts et al. (2012) reported that 86% of SUDEP cases are unwitnessed. It is hypothesized that being unaccompanied during a nocturnal seizure may carry even more risk than the severity of sleep-related respiratory dysfunction or PGES duration (Peng et al., 2017; Sveinsson et al., 2020). The presence of someone who could intervene and administer lifesaving resuscitative measures may mean the difference between a case of near SUDEP and actual SUDEP (Nashef et al., 1998; Langan et al., 2005; Lamberts et al., 2012). Increasing nocturnal supervision through the use of monitoring devices, checkups, or having another person asleep in the same room is associated with decreased SUDEP risk (Langan et al., 2005; Ryvlin et al., 2006; Harden et al., 2017). The majority of SUDEP victims are found prone in or near a bed (Opeskin and Berkovic, 2003; Sowers et al., 2013; Ali et al., 2017; Sveinsson et al., 2018). Many generalized seizures are followed by a period of PGES where the patient is more likely to be immobile, unresponsive, and require resuscitative measures (Semmelroch et al., 2012; Kuo et al., 2016). If a patient is unresponsive after a seizure that renders them prone, their nose and mouth may become obstructed by bedding. This may result in upper airway occlusion or asphyxiation against the surface the patient is positioned on. Outside of total airway occlusion, ending a seizure in the prone position on bedding may impairing postictal breathing by increasing inspiratory resistance and causing the patient to rebreathe trapped air (Kemp et al., 1994; Tao et al., 2010, 2015; Rugg-Gunn et al., 2016). This would cause an acute rise in CO_2_ in the blood, potentially leading to severe acidosis, which would potentiate the postictal immobility and further prolong the respiratory dysfunction until terminal apnea and asystole develop (Peng et al., 2017; Purnell et al., 2018).

### Clock genes

Although seizures are frequently thought to be unpredictable phenomenon, patients often display time-of-day-specific timing of seizure onset. In a recent study of patients implanted with responsive neurostimulators, it was shown that nearly 90% of patients with focal epilepsy had circadian timing of seizure onset (Leguia et al., 2021). Interestingly, circadian risk of seizure onset could be clustered into five general times of day, with seizures more likely to occur during the morning, mid-afternoon, evening, early night, or late night.

The circadian influence on seizures may be due in part to the bi-directional relationship of epilepsy and clock genes. Alterations in clock mechanisms increase the susceptibility for epilepsy, while seizures have the potential to disrupt the internal clock (Re et al., 2020). A significantly higher current is required to induce both maximal and generalized seizures in wild type (WT) mice during the dark phase of their diurnal cycle compared to the light phase. This rhythm is abolished in *Bmal1* KO mice, who also exhibit significantly lower seizure thresholds at all times compared to their WT counterparts (Gerstner et al., 2014). Similarly, conditional KO of *Bmal1* in neurons in the dentate gyrus increased the susceptibility to pilocarpine-induced seizures in mice (Wu et al., 2021). Hippocampal BMAL1 expression is reduced overtime in pilocarpine-treated rats as they begin to develop spontaneous seizures—suggesting that BMAL1 also plays a role in epileptogenesis (Matos et al., 2018). Levels of BMAL1 protein have been found to be decreased in the dentate gyrus and CA1 of mice with TLE (Wu et al., 2021). Mutations in the RAR related orphan receptor alpha (*RORA*) gene, which encodes for an activator of *Bmal1* transcription, have been linked to intellectual developmental disorder with or without epilepsy or cerebellar ataxia (IDDECA) (Guissart et al., 2018). Deletion of the gene *Clock* in cortical pyramidal neurons in mice results in epileptiform discharges in excitatory neurons as well as a decreased seizure threshold (Li et al., 2017). Real-time quantitative PCR (qPCR) analysis has revealed a loss in the rhythmic expression of CLOCK and decreased levels of its transcript in a post-status epilepticus rat model (Santos et al., 2015). *Clock* RNA and protein are similarly downregulated in brain tissue resected from patients with TLE (Li et al., 2017). Another oscillating clock gene, *Per1*, is upregulated in the hippocampus following electrical and kainic acid-induced seizures in mice (Eun et al., 2011). One study found an alteration in the rhythmic expression of PER1, PER2, and PER3 in a rat model of pilocarpine-induced seizures (Santos et al., 2015). However, a subsequent study found that an increase in PER1 expression and a decrease in PER2 expression prior to the development of spontaneous seizures, while PER3 expression was unaltered (Matos et al., 2018). To conclude, sleep and circadian phase have direct effects on periictal breathing and potentially the development of epilepsy itself.

## Sleep Impairment, Sleep-Disordered Breathing (SDB), and Epilepsy

### Sleep deprivation/sleep disorders

Apart from nocturnal seizures, patients with epilepsy also have a greater prevalence of sleep disorders compared to healthy individuals (Vaughn and D’cruz, 2004). A myriad of studies over the past 30 years have repeatedly found that adults with epilepsy are 2–3 times more likely to have a sleep/wake disorder compared to the general population (Grigg-Damberger and Foldvary-Schaefer, 2021). Patients with temporal lobe epilepsy exhibit reduced sleep efficiency and more arousals compared to those with frontal lobe epilepsy (Crespel et al., 2000). In addition, amygdala kindling decreases REM sleep in experimental animals, and selective REM sleep deprivation accelerates the kindling process (Cohen and Dement, 1970; Tanaka and Naquet, 1975). The *Scn1a^R1407X/+^* mouse shows impairments in circadian sleep regulation, including a fragmented rhythm of NREM sleep and an elongated circadian period of sleep (Sanchez et al., 2019).

Sleep deprivation caused by sleep disorders of frequent nocturnal seizures can result in sleep deprivation. Sleep deprivation itself can induce seizures and interictal spiking (Mattson et al., 1965; Pratt et al., 1968; Malow et al., 2000b; Konduru et al., 2021). In amygdala kindled cats, acute sleep deprivation reduces seizure and after discharge threshold (Shouse and Sterman, 1982). However, more prolonged sleep deprivation increases their susceptibility to both kindled and penicillin-induced seizures, regardless of sleep state (Shouse, 1988). Additionally, when kindled rats were administered a microinjection of a cholinergic agonist into the pontine reticular formation to enhance REM sleep, the result was a significant increase in the current threshold needed to elicit afterdischarge spiking in the amygdala (Kumar et al., 2007). Sleep deprivation studies in healthy individuals have shown hypertension and increased sympathetic nervous system activity after nights where sleep was less than 5 h (Lusardi et al., 1996; Tochikubo et al., 1996; Gangwisch et al., 2006). Thus, sleep deprivation may not only worsen seizures themselves, but also leave patients more vulnerable to seizure-induced autonomic insults.

### SDB

Up to 9–11% of adult patients with epilepsy exhibit SDB (Vendrame et al., 2014; Popkirov et al., 2019). This number jumps up to 40% when looking at children with epilepsy (Kaleyias et al., 2008). A recent case study highlighted a male patient with a history of secondary generalized tonic/clonic seizures who displayed paroxysmal nocturnal breathing. The patient experienced periods of breathing arrest in conjunction with an odd expiratory noise—primarily during REM sleep or the transition between REM and NREM—despite being seizure free for a year (Künstler et al., 2022).

OSA is a relatively common form of SDB, in which the upper airway collapses, preventing ventilation. The ensuing apnea provokes an arousal response which allows for re-positioning and recovery of gas exchange (Butler et al., 2015). The precise occurrence of OSA in people with epilepsy has yet to reach a consensus. Popkirov et al. estimates that 7% of epilepsy patients have mild-to-moderate OSA (Popkirov et al., 2019). A separate polysomnography study postulates that one-third of patients with medically refractory epilepsy who were candidates for epilepsy surgery have concomitant OSA (Malow et al., 2000b). This is also closer to an estimate produced from a meta-analysis in 2017, which determined the prevalence of mild-to-severe OSA in patients with epilepsy to be 33.4%—2.4 times more likely than healthy comparisons (Lin et al., 2017). This same meta-analysis found that the prevalence of OSA in patients with refractory epilepsy was not greater than the overall prevalence of OSA in patients with epilepsy (Lin et al., 2017). Patients with generalized epilepsy experience more severe OSA than those with focal epilepsy. Both populations reported similar degrees of abnormal daytime sleepiness. Older age, higher body mass index (BMI), and a history of hypertension are also associated with more severe OSA (Scharf et al., 2020). The incidence of OSA apnea in individuals without epilepsy is higher in males than in females (4% in men, 2% in women) (Block et al., 1979; Young et al., 1993). Men are also much more likely to experience O_2_ desaturation during apnea compared to women (Block et al., 1979). In patients with epilepsy, males are roughly three times more susceptible to OSA compared to females (Lin et al., 2017).

The length of obstructive apneas tends to increase over the course of a night’s sleep (Montserrat et al., 1996; Butler et al., 2015). It is suggested that this is due to a blunting of the CO_2_ arousal response over the course of the night, leading to longer periods of hypercapnia before arousal occurs (Montserrat et al., 1996). It is possible that this increase in OSA in epilepsy patients is due to an inherent blunting of chemosensitivity in an epileptic brain. Obese adolescents with OSA have an increased HCVR during wakefulness and a blunted HCVR during sleep (Yuan et al., 2012). There are also endogenous circadian components to the prolongation of respiratory events across the night. At circadian phases that correspond to the early morning, the duration of apnea and hypopneas are typically longer, but apnea/hypopnea index (AHI), a measurement of OSA severity, is low. In contrast, during the late afternoon to early evening, event durations were short and AHI was high. Events during REM sleep also tended to be 14% longer than those emerging from NREM sleep (Butler et al., 2015).

Comorbidity of epilepsy with OSA can increase the incidence of arrhythmias and increase the patient’s risk for sudden cardiac death (Gami and Somers, 2008; Gami et al., 2013). Patients with OSA experience disruption of the autonomic system during sleep (Adlakha and Shepard, 1998), which may be further imbalanced by seizures. While no direct correlation between OSA and SUDEP has been identified, higher revised SUDEP-7 scores [presence of seizures in the past 12 months—especially generalized tonic clonic seizures (GTCS), longer duration of epilepsy, increased number of ASMs, and lower IQ/more cognitive impairment]—are associated with probable SUDEP (Phabphal et al., 2021). OSA decreases the amount of time a person spends asleep each night, potentially leading to further sleep deprivation. Sleep deprivation is particularly dangerous for those with epilepsy as it can have an epileptogenic effect (Nobili et al., 2011; Popkirov et al., 2019). It follows then that when epilepsy patients with OSA were treated with continuous positive airway pressure (CPAP) they exhibited better seizure control than their untreated peers (Lin et al., 2017).

Vagus nerve stimulation (VNS) is a technique used to treat refractory epilepsy *via* a neurostimulation device. While these devices have been found to lessen seizure frequency and severity, there is a lack of conclusive evidence indicating that VNS lessens SUDEP risk (Annegers et al., 1998; Ryvlin et al., 2018). There is, however; evidence that VNS activation during sleep can induce mild OSA or exacerbate preexisting OSA. VNS activation during sleep is similarly linked to decreased V_T_ and SaO_2_, increased respiratory rate and AHI, and excessive daytime somnolence (Malow et al., 2000a; Holmes et al., 2003; Marzec et al., 2003; Zambrelli et al., 2016; Somboon et al., 2019; Kim et al., 2022). A recent study has also indicated HCVR slope is attenuated in patients with an active VNS (Sainju et al., 2021). Evidence suggests the exacerbation of OSA after VNS is due to reduction of the glottal space or lack of laryngeal–respiratory coordination (Zambrelli et al., 2016). This is notable as patients with refractory epilepsy are at higher risk for SUDEP and are more likely to opt for VNS as a method of seizure control. To summarize, individuals with epilepsy are more likely to experience sleep disorders and SDB, which may directly influence seizure frequency. Moreover, a common treatment for refractory epilepsy appears to aggravate SDB in these patients.

## Neurotransmitter Mechanisms

While the underlying mechanisms behind the sleep and circadian effects on breathing in epilepsy are still not fully understood, numerous neurotransmitters and signaling molecules have been implicated. For instance, the monoaminergic neurotransmitter serotonin (5-HT) plays an important role in sleep-wake regulation and respiration (Jouvet, 1999; Richerson, 2004; Hodges et al., 2009; Ptak et al., 2009; Hodges and Richerson, 2010; Depuy et al., 2011; Buchanan, 2013; Iwasaki et al., 2018; Smith et al., 2018). It is also heavily implicated in epilepsy and SUDEP pathophysiology (Bagdy et al., 2007; Richerson and Buchanan, 2011; Richerson, 2013; Feng and Faingold, 2017; Li and Buchanan, 2019; Petrucci et al., 2020). Serotonergic tone is modulated in both a sleep state and circadian phase-dependent manner, with the nadir occurring during the nighttime and during sleep (Mcginty and Harper, 1976; Rosenwasser et al., 1985; Agren et al., 1986; Rao et al., 1994; Sakai and Crochet, 2001; Mateos et al., 2009; Sakai, 2011; Purnell et al., 2018). 5-HT neurons in both the midbrain and medullary raphe have been demonstrated to be robustly chemosensitive (Larnicol et al., 1994; Richerson, 1995, 2004; Wang et al., 1998; Severson et al., 2003). It is likely that 5-HT neurons in the medulla mediate increased respiration in response to a rise in CO_2_ whereas midbrain 5-HT neurons mediate non-respiratory responses to CO_2_, such as arousal (Richerson, 2004; Buchanan and Richerson, 2010; Buchanan et al., 2015; Kaur et al., 2020). Firing of medullary raphe 5-HT neurons is markedly reduced during the ictal and postictal period, coinciding with severe respiratory depression (Zhan et al., 2016). Further, lower postictal serum 5-HT levels have been associated with postictal central apnea (Murugesan et al., 2019). Numerous studies have demonstrated that pre-treatment with serotonergic agents prior to seizure onset can ameliorate this respiratory dysfunction. The incidence of S-IRA in DBA/2 mice can be reduced through the administration of fluoxetine, a selective 5-HT reuptake inhibitor (SSRI), prior to seizure induction (Tupal and Faingold, 2019). A similar finding was discovered in DBA/1 mice, where fluoxetine was also found to reduce S-IRA without increasing basal ventilation or the ventilatory response to 7% CO_2_ (Zeng et al., 2015; Feng and Faingold, 2017). Other serotonergic agents, including fenfluramine, can selectively block S-IRA without influencing convulsive behavior (Feng and Faingold, 2017; Tupal and Faingold, 2019).

Another monoaminergic signaling molecule with links to sleep/wake regulation, respiration, and epilepsy is norepinephrine (NE; Hobson et al., 1975; Aston-Jones and Bloom, 1981; Foote et al., 1983). Plasma concentrations of NE are significantly lower during nocturnal sleep compared to wakefulness (Linsell et al., 1985). Like, 5-HT, NE also exhibits circadian rhythmicity with the lowest concentrations occurring during the night (Morgan et al., 1973; Agren et al., 1986; Cagampang and Inouye, 1994). The NE reuptake inhibitor (NRI) atomoxetine suppresses seizure-induced respiratory arrest following audiogenic seizures in DBA/1 mice (Zhang et al., 2017; Zhao et al., 2017) as well as MES seizures (Kruse et al., 2019). Another NRI, reboxetine, and the dual 5-HT/NE reuptake inhibitor (SNRI), duloxetine, are also able to suppress respiratory arrest following MES seizures (Kruse et al., 2019). More recently, evidence has indicated that selective activation of the noradrenergic α_2_ receptor is sufficient to suppress S-IRA in DBA/1 mice (Zhang et al., 2021).

The excitatory neuropeptide orexin is also involved in sleep and arousal and is a wake-promoting substance (Sakurai, 2007; Bonnavion and De Lecea, 2010; Nattie and Li, 2012). Orexin displays a strong diurnal circadian variation. This rhythm has been measured in the cerebral spinal fluid (CSF) and hypothalamus of rats, with an even stronger variation in the CSF of older rats (Yoshida et al., 2001; Desarnaud et al., 2004). This robust circadian rhythm is likely in part due to the dense projections that orexin neurons receive from the suprachiasmatic nucleus (SCN), the brain’s main circadian oscillator (Saper et al., 2005). Orexin neurons also contribute to respiratory function, in part due to orexinergic innervation of serotonergic and noradrenergic nuclei (Kuwaki, 2008; Inutsuka and Yamanaka, 2013). Orexin is thought to play a proconvulsant role in epilepsy, although there is some discrepancy regarding the effects of orexins and their antagonists on seizure activity. In *Kcna1*-null mutant mice, the dual orexin receptor antagonist (DORA), almorexant, decreases the incidence of severe seizures, improves O_2_ saturation, and increases overall longevity (Roundtree et al., 2016; Iyer et al., 2020).

The inhibitory neuromodulator, adenosine, is released in large quantities during seizures (During and Spencer, 1992; Berman et al., 2000; Van Gompel et al., 2014). This is likely a mechanism of seizure termination (Shen et al., 2010; Purnell et al., 2021a). Unlike, 5-HT, NE, and orexin, adenosine promotes sleep and suppresses wakefulness (Feldberg and Sherwood, 1954; Buday et al., 1961; Haulicǎ et al., 1973; Huber et al., 2004). As such, adenosine levels increase during wakefulness and are depleted during sleep (Porkka-Heiskanen et al., 2000; Bjorness and Greene, 2009). Adenosine has an inhibitory effect on respiration, predominately causing a reduction in frequency and V_T_ (Eldridge et al., 1984; Lagercrantz et al., 1984; Wessberg et al., 1984). The accumulation and clearance of adenosine is regulated in a circadian manner (Cornélissen et al., 1985; Chagoya De Sánchez et al., 1993; Huston et al., 1996). The adenosine hypothesis of SUDEP was first proposed in 2010, when Shen et al. noted that upregulated adenosine tone in a kainic acid model of epilepsy suppressed seizure activity but paradoxically caused death when seizures did occur (Shen et al., 2010). This hypothesis posits that a surge of adenosine is released during a seizure as a termination mechanism. However, this large increase in extracellular adenosine can result in suppression of breathing which can lead to terminal respiratory failure (Shen et al., 2010; Purnell et al., 2021a).

This is far from a comprehensive list of salient signaling molecules when it comes to respiratory function and SUDEP. However, these neuromodulators are especially of interest in the field of SUDEP and their complex role in sleep-wake regulation, breathing, and seizures makes them excellent candidates for therapeutic intervention.

## Conclusions

SUDEP is a complex and devastating phenomenon; the underlying mechanisms of which investigators are just beginning to unravel. The time of day and sleep state in which seizures occur are indisputably factors that can confer further risk to patients with epilepsy. While nighttime and sleep tend to go hand-in-hand, it is crucial that we acknowledge the two are not one in the same and come with their own risk factors from both shared and separate mechanisms. Respiratory failure is a major precipitating factor for seizure-induced death. Monoaminergic neurons, including 5-HT, NE, and orexin, play a crucial role in respiratory function and have seizure-protective properties. Levels of monoaminergic neurons are decreased during the nighttime and even further reduced during sleep. This may explain why seizures emerging from sleep tend to be longer and cause more severe respiratory dysfunction. Other signaling molecules, such as adenosine, may have an even more complex role in SUDEP pathophysiology—contributing to respiratory dysfunction during the process of terminating seizures. Many SUDEP victims are found in a prone position in bed, suggesting that respiratory distress was amplified by airway obstruction. As patients are also more likely to be unaccompanied at night, the chance of successful intervention is low.

Thus, while sleep and nighttime appear to confer their own risk of SUDEP, the fact that the two tend to occur in conjunction contributes greatly to the “perfect storm” of factors that ultimately leads to seizure-induced death. Nevertheless, it is our hope that this review imparts the notion that sleep state and time of day are factors that should be considered independently while developing preventative strategies to mitigate the severity of respiratory dysfunction brought about by seizures.

## Author Contributions

KJ and BK drafted the initial document. The final manuscript was edited and approved by KJ, BK, and GB. All authors contributed to the article and approved the submitted version.

## Funding

This published work was supported by National Institutes of Health/National Institute of Neurological Disorders and Stroke Grants F31NS125955 (to KJ), R01NS095842 (to GB), the Beth L. Tross Epilepsy Professorship from the Carver College of Medicine at the University of Iowa (to GB), and the National Institute of Health/National Institute of General Medicine Sciences T32GM0073367 (to BK).
